# Clinical outcomes of incident peritoneal dialysis patients coming from kidney transplantation program: A case-control study

**DOI:** 10.1371/journal.pone.0227870

**Published:** 2020-01-24

**Authors:** Laurisson Albuquerque da Costa, Maria Cláudia Cruz Andreoli, Aluizio Barbosa Carvalho, Sérgio Antonio Draibe, José Osmar Medina Pestana, Maria Eugênia Fernandes Canziani

**Affiliations:** Department of Internal Medicine, Division of Nephrology, Federal University of São Paulo, São Paulo, Brazil; University of Wisconsin, UNITED STATES

## Abstract

**Introduction:**

Brazil ranks second in the absolute number of transplantations in the world. Despite improvements in graft survival, many patients will progress to graft loss and return to dialysis. Concerns exist regarding adverse clinical outcomes in this population when undergone peritoneal dialysis (PD).

**Objective:**

To compare the occurrence of mortality, technique failure, and peritonitis among incident patients in PD coming from either Tx or pre-dialysis treatment.

**Methodology:**

A retrospective study in which 47 adult patients with Tx failure (Tx group) were matched for age, gender, diabetes mellitus (DM), modality and start year of PD, with 1:1 predialysis patient (nTx group). The Fine-Gray competing risk model was used to analyze mortality and technique failure.

**Results:**

Compared to nTx, the Tx group had a lower body mass index, serum potassium, and albumin concentrations. A higher ferritin level, transferrin saturation and the number of patients with positive serology for viral hepatitis were also observed in the Tx group. In the multivariate analysis, patients of the Tx group had 4.4-times higher risk of death (p = 0.007), with infection as the main cause. Technique failure and peritonitis were similar in both groups.

**Conclusion:**

Previous Tx is a risk factor for mortality but not for technique failure or peritonitis in incident patients on a PD program.

## Introduction

In Brazil, between 2007 and 2017, 54,790 kidney transplantations (Tx) were performed, and in 2016, the country ranked second in the absolute number of Tx in the world [1]. Despite advances in treatment and improvements in graft survival, many transplanted patients will progress to graft loss and require dialysis. In the United States, the probability of 5-year graft failure in 2011 was 25% for deceased-donor Tx and 15% for live-donor Tx [2]. In our center, the 10-year graft failure rate was 42.3% and 26.5%, respectively [3].

Tx failure is becoming a major cause of referring patients to dialysis [4]. Some studies have shown similar mortality among patients on dialysis after renal graft loss and never transplanted [5]. Peritoneal dialysis (PD), as an option for patients with renal graft loss, has been suggested as a triggering factor for unfavorable clinical outcomes [6]. However, some studies conducted in developed countries have shown that previous Tx is not a risk factor for survival among patients on PD [7] or for the occurrence of technique failure or peritonitis [8–10].

The objective of the present study, which was conducted in a single center in Brazil, is to compare the rates of mortality, technique failure, and peritonitis in incident patients on PD coming from either Tx or pre-dialysis treatment.

## Materials and methods

In this retrospective study, all patients who started PD after renal graft loss between January 2004 and June 2015 at the PD center of Hospital do Rim, Federal University of São Paulo, Brazil, were identified. Of the 60 patients identified, 13 were excluded due to: 1) having received more than 2 months of hemodialysis (HD) after graft failure (n = 5); 2) being on PD for less than 2 months (n = 3); 3) having more than one previous Tx (n = 3); 4) having undergone prior pancreas-kidney Tx (n = 1), and 5) being younger than 18 years old (n = 1). All patients underwent the standard training process before being admitted to the PD program.

Thus, 47 patients constituted the Tx group. These patients were matched for age, gender, the presence of diabetes mellitus (DM), PD modality [continuous ambulatory peritoneal dialysis (CAPD) or automated peritoneal dialysis (APD)] and the year of PD initiation with 47 patients on PD coming from pre-dialysis treatment [non-Tx group (nTx)]. None of the patients in the nTx group underwent prior Tx or dialysis. Clinical outcomes were collected until June 2016 or until the patients withdrew from the dialysis program.

Clinical and laboratory data at the beginning of the PD program were collected from patients’ files. The glomerular filtration rate was estimated by the CKD-EPI formula [11]. We also collected data on loss of residual renal function, being anuria considered as diuresis less than 100 mL within 24 hours. Clinical outcomes included data on death (number of deaths, time to death and cause), technique failure (number of events, time to failure and cause) and peritonitis (incidence density, etiologic agent and time to the first episode). Technique failure was defined as patient transfer to HD. The following circumstances were considered reasons for withdrawal from the study: renal function recovery, technique failure, transfer to another center, death or Tx.

The study was reviewed and approved by the Ethics Advisory Committee of the Federal University of São Paulo (approval number: 1.213.763, CAAE: 48094115.4.0000.5505) and the need for informed consent was waived. All data were anonymized to comply with the provisions of personal data protection legislation.

### Statistical analysis

The variables were presented as the mean ± standard deviation, median (interquartile range) or percentage depending on the characteristics of the variable. The groups were compared using the paired Student’s t-test, Wilcoxon test, chi-square test or Fisher’s test as appropriate. The Fine-Gray model was used to investigate the factors associated with the time to death and technique failure, considering death and technique failure as competing events. Cox proportional hazards model was used to investigate factors associated with the time to the first peritonitis episode. The models were fitted using simple and multiple approaches considering the variables of interest. Covariates with p < 0.05 in the univariate analysis were included in the multivariate model. The incidence density of peritonitis was estimated using a Poisson regression model considering the total follow-up time of each individual in the sample. P values < 0.05 were considered statistically significant. The analyses were performed using the statistical package R (R Core Team, 2017; Vienna, Austria), the survival (Therneau T, 2015 version 2.38, New York, United States) and cmprsk (Bob Gray, 2014, version 2.27) packages and IBM SPSS Statistics for Windows (version 20.0, Armonk, New York: IBM Corp).

## Results

The demographic and laboratory data of the two groups at the beginning of treatment are shown in Table 1. The groups were similar in age, gender, year of PD initiation, PD modality and presence of DM. Compared to the nTx, the Tx group had a lower BMI, a higher number of patients with positive serology for viral hepatitis (3 with hepatitis B, 3 with hepatitis C and 1 with both), lower concentrations of potassium and albumin and higher levels of ferritin and transferrin saturation.

**Table 1:** Demographic and laboratory characteristics at the beginning of the treatment.

	Tx	nTx	P
N	47	47	-
Age, years	46.1 ±12.1	47 ±12.7	0.74
Female gender	29 (62)	29 (62)	1.0
Start, 2004–2007 2008–2011 2012–2015	11 (23) 19 (40) 17 (36)	12 (26) 15 (32) 20 (43)	0.68
APD modality	40 (85)	40 (85)	1.0
Diabetes mellitus	15 (32)	15 (32)	1.0
White ethnicity	34 (72)	34 (72)	1.0
Education, high medium low	18 (38) 19 (40) 10 (21)	19 (40) 15 (32) 13 (28)	0.64
Etiology, Glomerulonephritis Diabetes mellitus Hypertension Unknown Other	15 (32) 6 (13) 7 (15) 10 (21) 9 (19)	10 (21) 12 (26) 6 (13) 10 (21) 9 (19)	0.09
Hypertension	41 (87)	45 (97)	0.27
Cardiovascular disease	16 (34)	14 (30)	0.66
Cancer	3 (6)	4 (9)	0.5
Viral hepatitis	7 (15)	0	0.01
BMI kg/m^2^	23 (20.4–24.9)	26.3 (22.8–29.9)	0.001
Presence of diuresis	40 (87)	44 (98)	0.11
Initial PET, medium low medium high low high	18 (56) 9 (28) 4 (13) 1 (3)	16 (42) 9 (24) 9 (24) 4 (11)	0.32
Urea, mg/dL	111.3 ±37.1	112.6 ±37.8	0.86
eGFR, mL/min/1.73 m^2^	6.0 (4.0–10)	8.2 (6.0–11.1)	0.08
Sodium, mEq/L	139.6±3.6	139.6±2.5	0.90
Potassium, mEq/L	4.2±0.6	4.5±0.5	0.01
Glucose, mg/dL	82 (77–106)	86 (78–99)	0.70
Hemoglobin, g/dL	10.5± 2	10.8±1.8	0.45
Leukocytes, cells/mm^3^	8105 ± 3047	7583 ± 2388	0.45
Ferritin, μg/L	303 (163–651)	157 (99.6–299)	0.001
Transferrin saturation, %	33.5 (22–42)	25 (21–33)	0.02
Phosphorus, mg/dL	5.8 ± 1.6	5.5 ±1.4	0.33
Calcium, mmol/L	1.2 (1.14–1.29)	1.25 (1.15–1.3)	0.20
iPTH, pg/dL	545 (192–904)	500 (283–807)	0.85
Albumin, g/dL	3.4 ±0.45	3.8 ±0.45	<0.001
Total cholesterol, mg/dL	196.2±49.2	197 ± 56	0.95
HDL, mg/dL	40 (33–51)	43 (35.5–52)	0.47
LDL, mg/dL	113.9±42.4	120.5±43.9	0.38
Triglycerides, mg/dL	154 (110–211)	148 (98–201)	0.23

The values are described as the mean ± standard deviation, median (interquartile range) or n (%); APD = automated peritoneal dialysis; PET = peritoneal equilibration test; eGFR = glomerular filtration rate estimated by the CKD-EPI equation; iPTH = intact parathyroid hormone; LDL = low-density lipoprotein; HDL = high-density lipoprotein

In the Tx group, the total time of previous renal replacement therapy (Tx + prior dialysis) was 120.5 (80–194.8) months, comprising 92.9 (49.9–165.4) months of kidney transplantation. The dialysis modalities performed before Tx were HD in 26 patients (55%), PD in 13 patients (28%) and both modalities in 5 (11%) patients. Pre-emptive Tx was performed in 3 (6%) patients. The PD follow-up was 14.8 (9.4–30.7) and 23.5 (2–21.2) months, in Tx and nTx groups, respectively (p = 0.42). During this period, 41 (87%) patients in the Tx group received 5.0 (3.3–5.0) mg/day of prednisone for a period of 10.9 (2–21.2) months. Among these patients, 1 patient also received cyclosporine and mycophenolate sodium, which was withdrawn during the first month, and 1 patient was maintained on tacrolimus and azathioprine for 3 months. No patient in the nTx group received corticosteroids or other immunosuppressive drugs. Eight (15%) patients underwent graft nephrectomy, 1 patient, before dialysis and 7 during the PD follow-up. During the observation period, the number of patients who underwent Tx was similar [13 (28%) vs 6 (13%); Tx and nTx groups; p = 0.12]. In addition, 1 patient (2%) within each group exhibited recovered renal function, whereas 3 patients (6%) and 1 (2%) were transferred to another center, in Tx and nTx groups, respectively.

**Fig 1:**
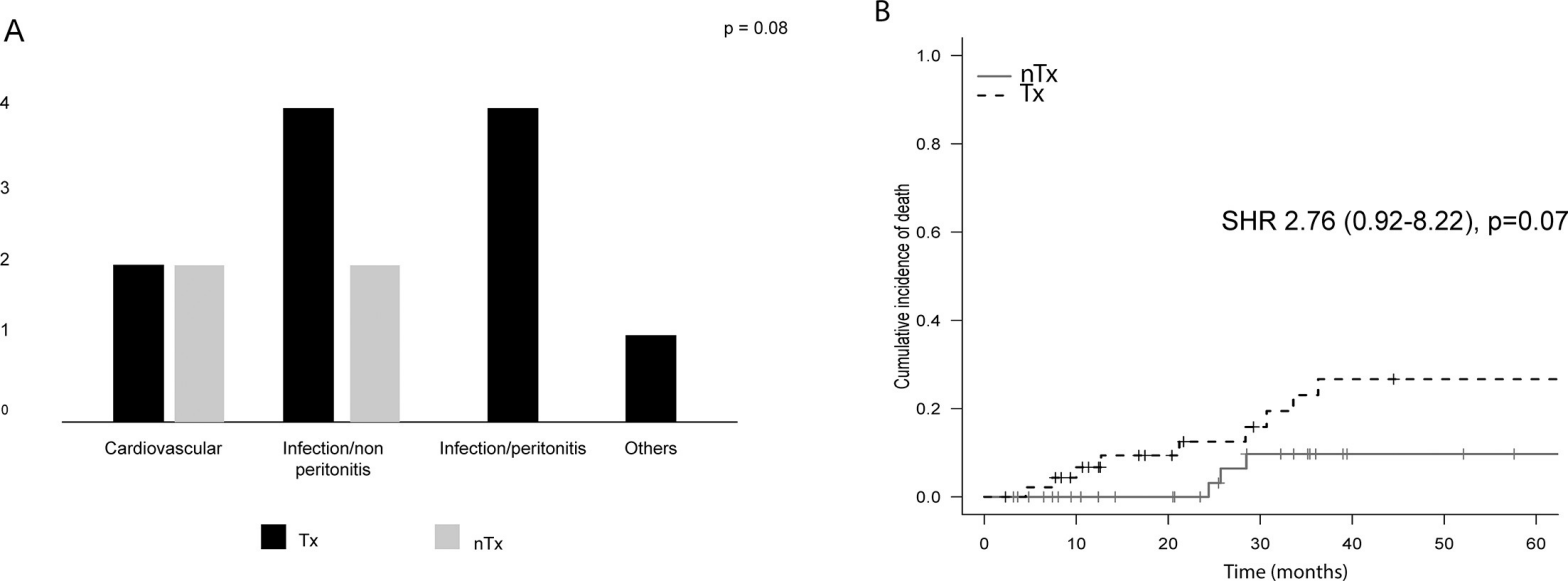
**Causes (A) and cumulative incidence (Fine-Gray model) (B) of death.** Tx = group with graft failure; nTx = group coming from pre-dialysis patients; SHR = subdistribution hazard ratio.

A trend toward a higher number of deaths in the Tx group was observed [11 (23%) vs 4 (9%), p = 0.09]. The causes of death are shown in Fig 1A. There was a trend toward a higher number of deaths due to infectious causes in the Tx group (p = 0.08). Fig 1B shows the cumulative incidence of death in the univariate model. Considering the competitive risk with technique failure there was a trend toward a higher risk of death in the Tx group [subdistribution hazard ratio (SHR) 2.76 (95% CI 0.92–8.22), p = 0.07]. However, in the multivariate model adjusted for age and urea, as shown in Table 2, the individuals with previous Tx had a higher risk of death [SHR = 4.4 (95% CI 1.49–13.2), p = 0.007].

**Table 2:** Multivariate analysis: Death and technique failure as competing events (Fine-Gray model).

Fine Gray to Death SHR (95%CI)		p	Fine Gray to technique failure SHR (95%CI)		p
Previous Transplantation	4.44 (1.49; 13.22)	**0.007**	Previous Transplantation	1.14 (0.59; 2.21)	0.69
Age (years)	1.09 (1.03; 1.15)	**0.002**	Glucose (10 units)	1.06 (1.01; 1.11)	**0.03**
Urea	0.99 (0.97; 0.998)	**0.03**	Urea	1.00 (0.997; 1.001)	0.33
			Phosphorus	1.260 (1.02; 1.56)	**0.04**

**Fig 2:**
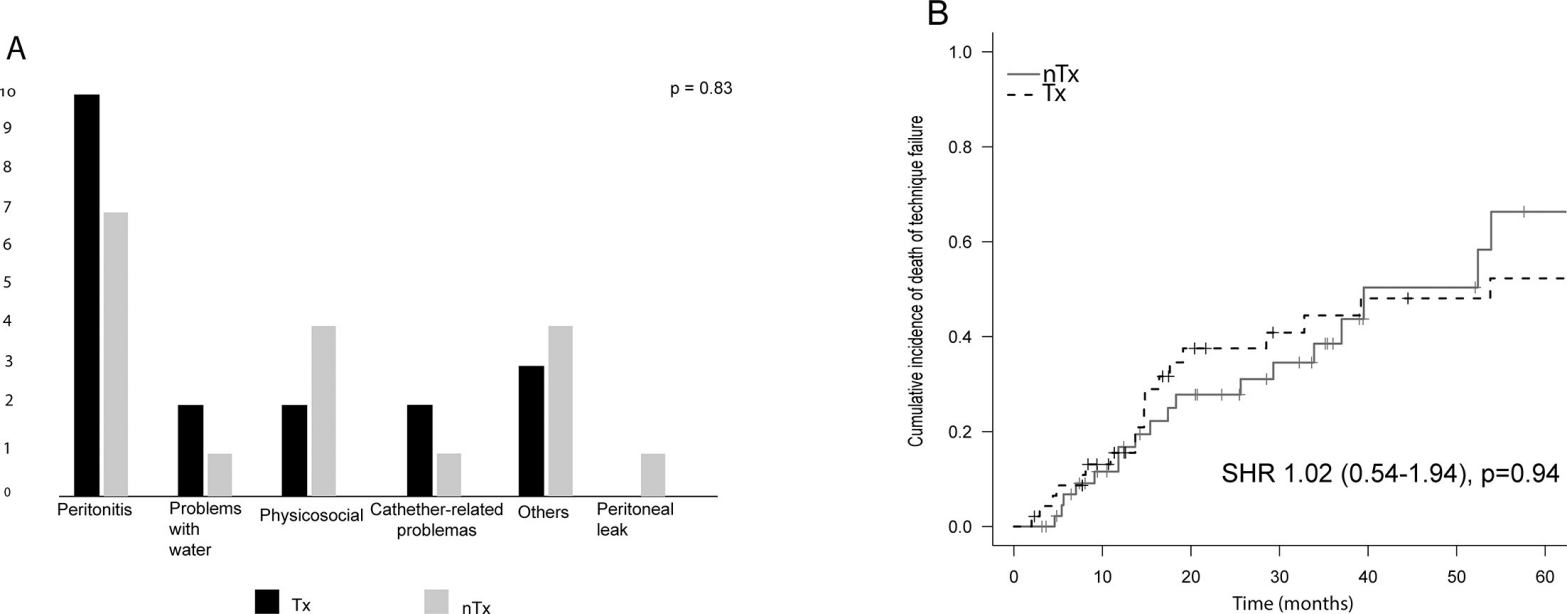
**Causes (A) and cumulative incidence (Fine-Gray model)(B) of technique failure.** Tx = group with graft failure; nTx = group coming from pre-dialysis patients; SHR = subdistribution hazard ratio.

The number of patients with technique failure was similar between the groups [19 (40%) vs 18 (38%), Tx and nTx groups, respectively; p = 0.83]. The main cause of technique failure in both groups was peritonitis (Fig 2A). In addition, Fig 2B shows that the cumulative incidence of technique failure without adjustments was similar between both groups [SHR 1.02 (95% CI 0.54–1.93), p = 0.94]. In the multivariate model for technique failure adjusted for glucose, urea and phosphorus, as shown in Table 2, previous Tx was not associated with the occurrence of technique failure [SHR 1.14 (95% CI 0.59–2.21), p = 0.69].

**Fig 3:**
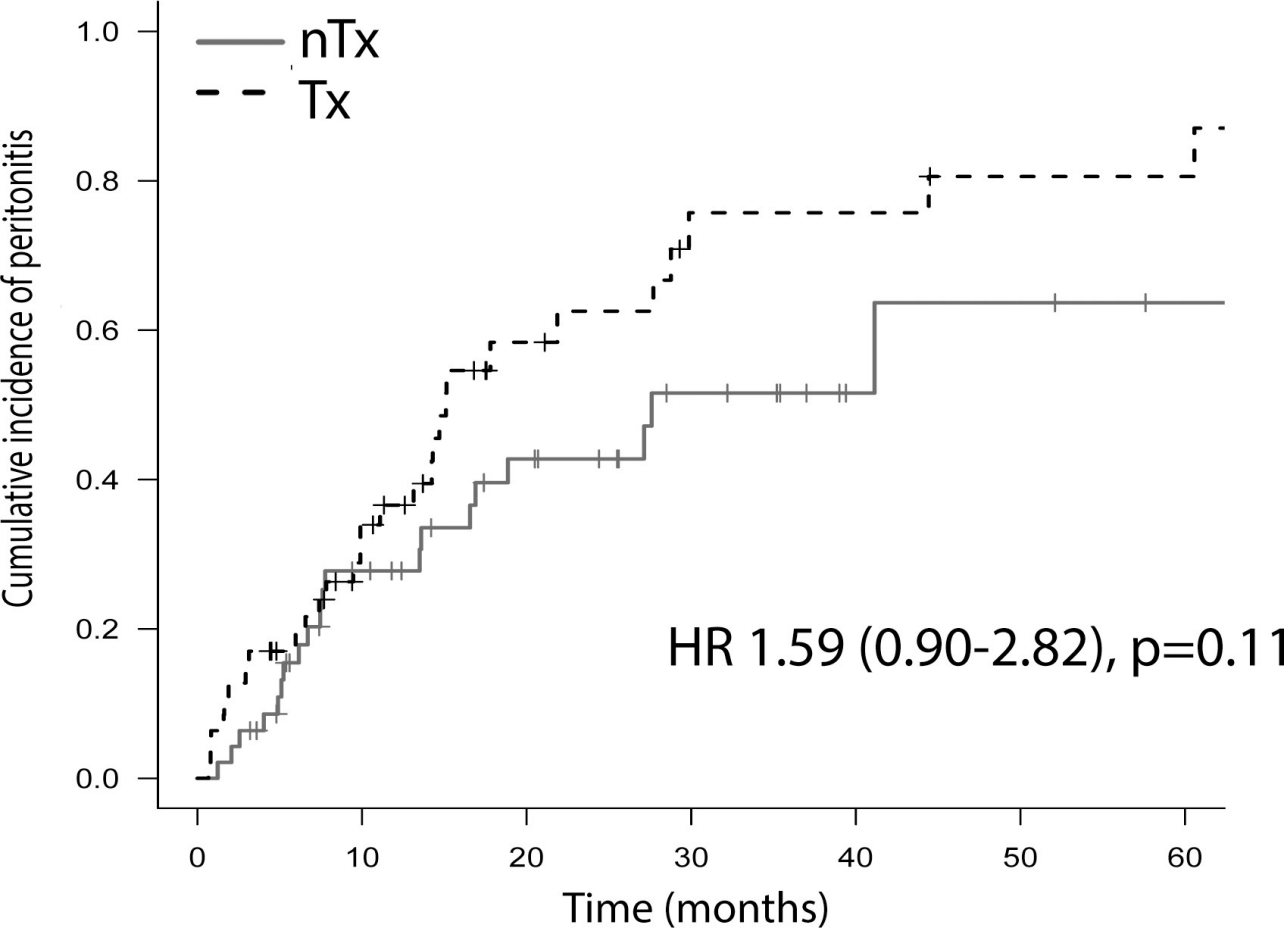
Cumulative incidence of peritonitis (Cox model). Tx = group with graft failure; nTx = group coming from pre-dialysis patients; HR = Hazard ratio.

When the groups were compared, the number of patients with peritonitis was not different [29 (62%) vs 20 (43%), Tx and nTx groups, respectively; p = 0.63]. A trend toward a higher incidence density of peritonitis was identified in the Tx group (0.59 vs 0.40 episodes/patient/year, Tx and nTx groups, respectively; p = 0.06). The time to the first peritonitis episode was similar between the groups [9.9 (3.0–6.5) vs 7.5 (5.0–16.8) months, Tx and nTx groups, respectively; p = 0.73]. Fig 3 shows the cumulative incidence of peritonitis, which was similar between the groups [HR 1.59 (95% CI 0.90–2.82); p = 0.11]. In the multivariate model adjusted for albumin, prior Tx was not a risk factor for peritonitis [HR 1.41 (95% CI 0.78–2.56); p = 0.25].

No difference was found in the microorganisms identified in the peritoneal dialysate between the two groups (p = 0.68). Gram-positive agents were found more frequently, and among these agents, *Staphylococcus aureus* had the highest incidence (Table 3).

**Table 3:** Microbiological characteristics of peritonitis.

Microorganism	Tx	nTx
**Gram-positive cocci**	**28 (50)**	**18 (47)**
*Staphylococcus aureus*	17 (30)	10 (26)
Coagulase-negative	8 (14)	7 (18)
Other	3 (5)	1 (3)
**Gram-negative bacilli**	**9 (16)**	**5 (13)**
*Pseudomonas*	4 (7)	1 (3)
**Fungi**	1 (2)	0
**Gram-positive bacilli** (*Corynebacterium* spp.)	0	1 (3)
**Other (not identified)**	7 (13)	5 (13)
**Negative culture**	11 (20)	9 (24)

n (%)

After one year in PD program 21 and 22 patients from Tx and nTx groups, respectively, had data on diuresis recorded in their files. From these, 17 (81%) in Tx and 2 (9%) in nTx group evolved to anuria (p<0,001).

## Discussion

The present study showed that patients who started PD after Tx failure presented a higher risk of death compared to those coming from pre-dialysis treatment. By contrast, the two groups did not differ regarding the occurrence of technique failure or peritonitis.

The impact of previous Tx on the survival of the PD population remains controversial in the literature. Similar to our findings, one study [6] reports worse survival rates among patients on PD with previous Tx, while other studies do not confirm this relationship [7–9, 12, 13]. These divergent results could be partially related to the characteristics of the studied population. Some studies did not control the presence of diabetes or even the age of the patients, which are well-known risk factors for mortality [14, 15]. Other factors such as pre-dialysis care, albumin, hemoglobin, residual diuresis and glomerular filtration rate have been associated with mortality in patients undergoing dialysis after graft loss [16]. In the present study, Tx patients had higher mortality mainly due to infectious diseases, which could be partially explained by the lower albumin levels and BMI observed in this group on the time of admission in the PD program. These findings could be a characteristic of the Tx failure phase. In fact, this period has been frequently associated with low serum albumin, elevated inflammation markers, and poor nutritional status [17], partially due to poor clinical management provided during the progressive graft loss period [18–20]. Of note, low albumin is described as a risk factor to the occurrence of peritonitis and mortality from infectious causes in PD cohorts [21, 22].

Another factor as iron overload, evidenced by higher serum ferritin and transferrin saturation, could also have contributed to the high mortality rate in the Tx group. Some studies involving HD patients with intravenous iron supplementation have shown that iron overload is associated with a higher occurrence of infectious disease [23–25]. This relationship has not yet been widely studied in PD patients. The higher mortality rate observed in the Tx group could also be related to the loss of residual renal function. In a subset of the studied population, a higher number of patients from the Tx group evolved with anuria, which can be explained by the fast reduction of immunosuppressive drugs. Studies have demonstrated that the greater loss of kidney function is related to worse survival in the PD population [26, 27]. Although the loss of residual kidney function has been more frequent in PD patients with previous Tx [28], the direct relationship between that condition and mortality in this population is not clear [29]. One could speculate why the cause of mortality in our patients was infectious, while US data from mortality in PD population point out to cardiovascular disease [2]. One hypothesis could be the characteristic of the study design, as 50% of the patients came from the Tx program. Differently from patients without previous transplant, these patients were exposed to an immunosuppressive regimen.

Regarding the technique survival, in accordance with others [10, 29], no difference was found between the groups in the present study. In contrast, Chaudhri et al. found a higher rate of technique failure in patients coming from Tx compared to never-transplanted patients [12]. Yet Benomar et al. have reported similar results, and pointed out that poor dialysis adequacy was the main cause of technique failure [7]. Of note, in the present study, peritonitis was the main cause of technique failure in both groups.

Considering peritonitis, Chen et al. found a trend toward a lower event-free time in pediatric patients who started PD after Tx failure compared to those who were transplanted naïve [30]. However, studies on the adult population showed no difference regarding peritonitis between groups [7, 9, 12]. Notably, in those studies, different immunosuppressive protocols were performed after PD initiation, making a comparison between them difficult to analyze. In the present study, a trend toward a higher incidence of peritonitis in the Tx group was found, which could be related to the use of immunosuppressive drugs by these patients.

The present study has some limitations, including the relatively small sample and the single-center retrospective design. It should also be noted that the Tx group has had a longer CKD time, and consequently, longer exposure to disease complications. However, our sample is one of the largest cohorts among single-center studies in patients with graft failure starting PD. In addition, we employed appropriate statistical analyses as a competitive risk for the main outcomes.

In conclusion, previous Tx is a risk factor for mortality but not for technique failure or peritonitis in incident patients on a PD program. Further studies with larger samples are needed and also could be of interest to investigate the outcomes of incident patients on HD program coming from Tx, to establish a better dialysis modality, PD or HD, and to better management of immunosuppressives drugs to patients after kidney allograft failure.

## Supporting information

S1 Appendix All data. (XLS)
